# Comparative analysis of codon usage patterns of *Plasmodium* helical interspersed subtelomeric (PHIST) proteins

**DOI:** 10.3389/fmicb.2023.1320060

**Published:** 2023-12-14

**Authors:** Baoling Yang, Ziwen Cheng, Like Luo, Kuo Cheng, Shengqi Gan, Yuyi Shi, Che Liu, Dawei Wang

**Affiliations:** ^1^College of Basic Medicine, Jinzhou Medical University, Jinzhou, Liaoning Province, China; ^2^College of Animal Husbandry and Veterinary Medicine, Jinzhou Medical University, Jinzhou, Liaoning Province, China

**Keywords:** *Plasmodium falciparum*, *Plasmodium* helical interspersed subtelomeric proteins, codon usage bias, relative synonymous codon usage, effective number of codons

## Abstract

**Background:**

*Plasmodium falciparum* is a protozoan parasite that causes the most severe form of malaria in humans worldwide, which is predominantly found in sub-Saharan Africa, where it is responsible for the majority of malaria-related deaths. *Plasmodium* helical interspersed subtelomeric (PHIST) proteins are a family of proteins, with a conserved PHIST domain, which are typically located at the subtelomeric regions of the *Plasmodium falciparum* chromosomes and play crucial roles in the interaction between the parasite and its human host, such as cytoadherence, immune evasion, and host cell remodeling. However, the specific utilization of synonymous codons by PHIST proteins in *Plasmodium falciparum* is still unknown.

**Methods:**

Codon usage bias (CUB) refers to the unequal usage of synonymous codons during translation, resulting in over- or underrepresentation of certain nucleotide patterns. This imbalance in CUB can impact various cellular processes, including protein expression levels and genetic variation. To investigate this, the CUB of 88 PHIST protein coding sequences (CDSs) from 5 subgroups were analyzed in this study.

**Results:**

The results showed that both codon base composition and relative synonymous codon usage (RSCU) analysis identified a higher occurrence of AT-ended codons (AGA and UUA) in PHIST proteins of *Plasmodium falciparum*. The average effective number of codons (ENC) for these PHIST proteins was 36.69, indicating a weak codon preference among them, as it was greater than 35. Additionally, the correlation analysis among codon base composition (GC1, GC2, GC3, GCs), codon adaptation index (CAI), codon bias index (CBI), frequency of optimal codons (FOP), ENC, general average hydropathicity (GRAVY), aromaticity (AROMO), length of synonymous codons (L_sym), and length of amino acids (L_aa) revealed the influence of base composition and codon usage indices on codon usage bias, with GC1 having a significant impact in this study. Furthermore, the neutrality plot analysis, PR2-bias plot analysis, and ENC-GC3 plot analysis provided additional evidence that natural selection plays a crucial role in determining codon bias in PHIST proteins.

**Conclusion:**

In conclusion, this study has enhanced our understanding of the characteristics of codon usage and genetic evolution in PHIST proteins, thereby providing data foundation for further research on antimalarial drugs or vaccines.

## Introduction

Proteins are the primary bearers of life activities, composed of codons made up of nucleotides that are translated into amino acids. Therefore, the final function of a protein is influenced by the usage of its codons. Proteins are primarily composed of 20 standard amino acids, and different species use various synonymous codons to encode the 18 amino acids except methionine (Met) and tryptophan (Trp) (Parvathy et al., 2022; Jiang et al., 2023). Although the genetic code has evolved, it remains highly conserved, allowing for the use of different codons to encode the same amino acid (Chaney and Clark, 2015; Bailey et al., 2021). The usage of synonymous codons is not uniform or predictable across organisms, genes, or even within the same gene in different species. Certain codons are often favored for encoding specific amino acids (Pakrashi et al., 2023; Wang et al., 2023; Zhao et al., 2023). Codon Usage Bias (CUB) refers to the phenomenon where synonymous codons are used with varying frequencies (Iriarte et al., 2021; Alqahtani et al., 2022; Chen and Yang, 2022; Khandia et al., 2022). These synonymous mutations, also known as “silent mutations,” do not alter the original protein sequence or structure. However, variations in synonymous codons among organisms can significantly contribute to genome evolution. Previous studies have identified several factors that influence CUB in different organisms, with natural selection (e.g., translation, gene length, and gene function) and mutation pressure (e.g., GC content and mutation position) being considered as the fundamental factors (Bhattacharyya et al., 2019; Shen G. et al., 2022; Shen X. et al., 2022; Hu et al., 2023; Matsushita and Kano-Sueoka, 2023). CUB has a significant impact on various cellular processes, including mRNA stability, transcription, translation efficiency and accuracy, protein structure, folding, expression, and function. Understanding CUB has practical applications in heterologous gene expression, species identification, primer design, predicting gene expression levels and functions, as well as designing synthetic genes for biotechnological purposes (Gorlov et al., 2018; Chassalevris et al., 2020; Wang and Blenner, 2022; Hernandez-Alias et al., 2023; Vaz et al., 2023). However, most studies on CUB have focused on bacteria, fungi, viruses, and mycoplasma (Dilucca et al., 2020; Hou, 2020; Wu et al., 2021; Li et al., 2022). Currently, there is still a lack of comprehensive understanding regarding the genetic features of codon bias in parasites, particularly in *Plasmodium falciparum*.

The World Malaria Report 2022 indicates that there were 247 million cases of malaria globally in 2021, with an estimated 619,000 deaths. Cases are mainly concentrated in certain countries and regions in Africa, Asia, and the Americas, so global malaria control still faces many challenges. Over the past 20 years, the development and large-scale promotion of rapid diagnostic tests (RDTs), artemisinin-based combination therapy (ACT), and insecticide-treated nets (ITN) have been key to the global successful response to malaria (WHO, 2022). *Plasmodium falciparum* goes through various stages in its lifecycle, including the injection of sporozoites into the bloodstream by mosquitoes. These sporozoites invade liver cells and multiply, producing merozoites. The merozoites are released into the bloodstream and infect red blood cells. Inside the red blood cells, the parasites multiply and eventually cause the cells to rupture, releasing more merozoites to infect other red blood cells. Multiple studies have identified various invasion-related protein molecules mediating the invasion process of *Plasmodium falciparum*, such as *Plasmodium falciparum* erythrocyte membrane protein 1 (PfEMP1), *Plasmodium falciparum* merozoite surface protein 1 (PfMSP1), *Plasmodium falciparum* apical membrane antigen 1 (PfAMA1), *Plasmodium falciparum* rhoptry neck protein 4 (PfRON4), with PfEMP1 being particularly important (Lee et al., 2023; Leonard et al., 2023; Pulido-Quevedo et al., 2023; Wiser, 2023). In recent years, the involvement of *Plasmodium* helical interspersed subtelomeric (PHIST) proteins in the invasion process has also been investigated. PHIST is a unique protein family of *Plasmodium falciparum* and the PHIST proteins are characterized by a conserved domain of approximately 150 amino acids that are predicted to form four consecutive alpha helices. Some members of this family consist of an export signal sequence, the PEXEL motif, and the PHIST domain, while others also contain a DnaJ domain and tryptophan residue (Fierro et al., 2023; Hasan et al., 2023). Besides, the PHIST family includes different subgroups, such as PHISTa, PHISTa-like/PHIST, PHISTb, PHISTb-DnaJ, and PHISTc, which plays crucial roles in the parasite’s biology, including host cell invasion, immune evasion, and modulation of the host immune response (Warncke et al., 2016; Kumar et al., 2019; Mutisya et al., 2020; Yang et al., 2020). Although the functional domains of PHIST proteins are conserved, the coding sequences vary significantly, and there is limited research on their codon usage. To gain insights into the genetics and evolution of *Plasmodium falciparum*, as well as to predict the function and regulation mechanisms of related genes, we conducted a systematic analysis and comparison of CUB among 88 sequences of PHIST proteins from five subgroups. We constructed a phylogenetic tree based on the relative synonymous codon usage and compared it with the tree constructed using the CDSs of PHIST proteins. This analysis of CUB provides further understanding of the *Plasmodium falciparum*’s genetic characteristics and evolution.

## Materials and methods

### Sequences

A total of 88 PHIST protein CDSs from *Plasmodium falciparum* were retrieved from the PlasmoDB^1^ for CUB analysis. The 88 PHIST proteins were divided into 5 subgroups (PHISTa, PHISTa-like/PHIST, PHISTb, PHISTb-DnaJ, PHISTc) and detailed information about these proteins is listed in Table 1.

**Table 1:** Codon usage indices in PHIST proteins.

Subgroup	ID	CAI	CBI	FOP	ENC	GRAVY	AROMO	L_sym	L_aa
PHISTa	PF3D7_0102000	0.164	−0.279	0.272	37.42	−0.452	0.147	243	251
	PF3D7_0115100	0.150	−0.225	0.309	33.53	−0.844	0.102	288	293
	PF3D7_0402000	0.192	−0.152	0.368	36.61	−1.459	0.061	419	428
	PF3D7_0424900	0.161	−0.224	0.305	40.11	−0.801	0.092	279	283
	PF3D7_0425300	0.146	−0.283	0.260	34.17	−0.409	0.108	223	232
	PF3D7_0425400	0.167	−0.274	0.288	41.80	−0.806	0.126	226	231
	PF3D7_0601700	0.151	−0.203	0.314	38.99	−0.833	0.104	280	288
	PF3D7_0800600	0.146	−0.233	0.301	33.13	−0.804	0.096	286	291
	PF3D7_0831750	0.174	−0.130	0.346	34.12	−0.640	0.101	283	287
	PF3D7_1000700	0.158	−0.221	0.304	37.36	−0.421	0.140	276	286
	PF3D7_1001100	0.177	−0.180	0.329	36.95	−0.846	0.077	307	313
	PF3D7_1001300	0.159	−0.272	0.275	34.31	−0.619	0.115	269	278
	PF3D7_1100600	0.179	−0.188	0.328	36.21	−0.673	0.118	271	279
	PF3D7_1149700	0.138	−0.383	0.125	31.88	−0.591	0.176	80	85
	PF3D7_1253100	0.164	−0.217	0.314	31.48	−0.763	0.132	280	288
	PF3D7_1253300	0.144	−0.243	0.280	38.81	−0.560	0.107	200	205
	PF3D7_1253800	0.168	−0.253	0.294	34.61	−0.717	0.123	279	284
	PF3D7_1253900	0.152	−0.200	0.316	36.91	−0.542	0.106	269	274
	PF3D7_1301100	0.157	−0.212	0.310	36.72	−0.581	0.116	271	276
	PF3D7_1301500	0.148	−0.297	0.271	35.43	−0.669	0.136	292	308
	PF3D7_1372000	0.177	−0.232	0.316	30.99	−1.118	0.108	408	417
	PF3D7_1400900	0.162	−0.197	0.321	36.75	−0.575	0.116	271	276
	PF3D7_1477700	0.153	−0.185	0.317	41.07	−0.669	0.100	281	291
	PF3D7_1478000	0.190	−0.218	0.318	39.01	−0.790	0.135	274	282
	PF3D7_1478500	0.125	−0.315	0.243	32.55	−0.486	0.103	251	262
	PF3D7_1479200	0.169	−0.249	0.294	35.08	−0.767	0.120	279	284
	PF3D7_1479300	0.157	−0.212	0.310	36.72	−0.581	0.116	271	276
PHISTa-like/PHIST	PF3D7_0425250	0.160	−0.167	0.337	44.25	−0.856	0.141	172	177
	PF3D7_0831300	0.201	−0.260	0.308	35.84	−1.426	0.104	789	821
	PF3D7_0831500	0.141	−0.354	0.245	30.55	−0.945	0.099	327	335
	PF3D7_0831900	0.174	−0.217	0.312	44.57	−0.650	0.095	276	284
	PF3D7_0832200	0.165	−0.242	0.296	34.90	−0.670	0.112	277	286
	PF3D7_0832300	0.141	−0.284	0.267	39.19	−0.500	0.120	251	259
	PF3D7_0832700	0.133	−0.299	0.256	32.55	−0.520	0.136	246	257
	PF3D7_1201200	0.129	−0.239	0.284	35.93	−0.649	0.081	215	221
	PF3D7_1301300	0.176	−0.186	0.336	34.29	−0.916	0.111	238	244
	PF3D7_1372300	0.140	−0.244	0.279	39.93	−0.536	0.112	201	206
	PF3D7_1477300	0.177	−0.156	0.338	47.25	−0.513	0.121	272	280
	PF3D7_1477400	0.165	−0.213	0.304	39.07	0.038	0.165	289	303
PHISTb	PF3D7_0201600	0.174	−0.224	0.310	38.54	−0.930	0.136	468	485
	PF3D7_0401800	0.154	−0.412	0.227	30.90	−0.604	0.068	550	560
	PF3D7_0402100	0.182	−0.209	0.318	39.36	−0.821	0.115	556	575
	PF3D7_0424600	0.193	−0.105	0.376	37.99	−0.616	0.117	295	309
	PF3D7_0424800	0.130	−0.230	0.287	35.73	−0.533	0.083	307	314

	PF3D7_0532300	0.211	−0.106	0.376	37.06	−0.945	0.096	487	509
	PF3D7_0532400	0.179	−0.173	0.339	34.66	−0.949	0.089	513	528
	PF3D7_0601500	0.156	−0.284	0.275	34.96	−0.693	0.109	448	467
	PF3D7_0631100	0.157	−0.287	0.273	35.09	−0.662	0.109	447	466
	PF3D7_0702100	0.160	−0.189	0.324	36.15	−0.755	0.083	655	671
	PF3D7_0731300	0.162	−0.268	0.275	32.78	−0.608	0.117	305	317
	PF3D7_0831000	0.166	−0.260	0.291	34.31	−0.649	0.138	409	426
	PF3D7_0902700	0.231	−0.107	0.390	35.08	−1.151	0.098	433	447
	PF3D7_0936900	0.182	−0.165	0.339	37.74	−0.776	0.122	351	362
	PF3D7_0937000	0.173	−0.234	0.299	37.22	−0.508	0.146	341	357
	PF3D7_1102500	0.173	−0.171	0.337	36.80	−0.813	0.111	602	624
	PF3D7_1201000	0.274	−0.075	0.405	38.12	−1.351	0.074	644	653
	PF3D7_1252700	0.151	−0.258	0.289	35.50	−0.703	0.104	436	450
	PF3D7_1252800	0.143	−0.278	0.285	35.73	−1.004	0.099	600	626
	PF3D7_1372100	0.168	−0.204	0.316	39.16	−0.845	0.115	588	609
	PF3D7_1401600	0.205	−0.147	0.355	38.38	−0.824	0.103	456	478
	PF3D7_1476200	0.211	−0.172	0.357	37.07	−1.518	0.086	502	512
	PF3D7_1476300	0.209	−0.146	0.359	36.23	−1.031	0.103	555	572
PF3D7_1477500	0.185	−0.102	0.372	40.64	−0.999	0.124	489	500
PHISTb-DnaJ	PF3D7_0102200	0.239	−0.033	−0.416	36.65	−0.944	0.083	1,057	1,085
	PF3D7_0201700	0.169	−0.171	0.336	38.03	−0.718	0.093	870	900
	PF3D7_0220100	0.195	−0.142	0.356	38.96	−0.847	0.098	962	997
	PF3D7_1038800	0.153	−0.237	0.300	36.76	−0.758	0.101	877	911
	PF3D7_1149200	0.195	−0.098	0.381	35.73	−0.939	0.077	1,059	1,090
	PF3D7_1149500	0.165	−0.152	0.344	39.56	−0.682	0.112	816	838
	PF3D7_1201100	0.167	−0.205	0.319	35.35	−0.792	0.118	856	900
PHISTc	PF3D7_0202100	0.153	−0.284	0.270	33.48	−0.572	0.099	282	302
	PF3D7_0219700	0.174	−0.222	0.311	36.26	−0.778	0.115	280	295
	PF3D7_0219800	0.182	−0.119	0.373	49.72	−0.861	0.132	303	319
	PF3D7_0424000	0.158	−0.179	0.331	44.01	−1.008	0.089	341	347
	PF3D7_0532200	0.181	−0.231	0.307	45.83	−0.686	0.124	212	226
	PF3D7_0731100	0.149	−0.292	0.286	34.42	−1.133	0.126	866	890
	PF3D7_0801000	0.131	−0.287	0.274	33.16	−1.550	0.099	1,182	1,219
	PF3D7_0830600	0.157	−0.281	0.276	33.75	−0.904	0.121	442	456
	PF3D7_0936600	0.156	−0.204	0.309	36.10	−0.561	0.104	288	297
	PF3D7_0936800	0.184	−0.141	0.356	35.07	−1.167	0.084	371	383
	PF3D7_1001700	0.209	−0.167	0.346	35.52	−0.959	0.120	257	266
	PF3D7_1001800	0.184	−0.223	0.315	34.66	−0.746	0.092	251	260
	PF3D7_1016500	0.160	−0.242	0.315	38.81	−1.170	0.116	739	767
	PF3D7_1016600	0.183	−0.234	0.314	29.78	−0.830	0.162	280	296
	PF3D7_1016700	0.163	−0.309	0.283	36.95	−1.293	0.125	799	830
	PF3D7_1016800	0.208	−0.090	0.383	35.26	−1.048	0.134	298	314
	PF3D7_1148700	0.191	−0.100	0.384	35.70	−0.919	0.078	359	370
	PF3D7_1200900	0.166	−0.340	0.260	32.53	−0.874	0.149	366	383

The codon adaptation index (CAI), codon bias index (CBI), frequency of optimal codons (FOP), the effective number of codons (ENC), general average hydropathicity (GRAVY), aromaticity (AROMO), length of synonymous codons (L_sym) and length of amino acids (L_aa) were calculated in PHIST proteins by CodonW software, respectively. Single underline represents the minimum value; Double underline represents the maximum value.

### Analysis of codon base composition

The codon base composition of 88 PHIST proteins was determined by CodonW software. Only 59 synonymous codons were analyzed in this study, except the first AUG codon (Met), the codon (UGG) encoding Trp, and the three termination codons (UAG, UAA, and UGA), respectively. The nucleotide at the 3rd codon location (C3, T3, G3, and A3%), the GC% contents of all three codon locations (GC1, GC2, and GC3%) and total GCs% and ATs% contents were measured (Boissinot, 2022).

### Analysis of codon usage indices

Multiple factors can have an impact on CUB, thus, many statistical methods have been proposed to analyze the codon usage indices. The codon adaptation index (CAI) is a measure used to quantify the similarity between the codon usage of a gene or sequence and the codon usage bias of a reference set, which calculates the frequency of occurrence of each codon in the gene of interest and compares it to the frequency expected based on the codon usage bias observed in a reference set, typically a set of highly expressed genes in the same organism. And the CAI value ranges from zero to one; the larger the value is, the more frequently the CUB (Zhou et al., 2023). The codon bias index (CBI) reflects the components of highly expressed superior codons in a specific gene. And the value of CBI near zero indicates all codons are completely randomly used (Masłowska-Górnicz et al., 2022). The frequency of optimal codons (FOP) ranges from 0.36 (which means the codon usage bias is weak) to 1 (which means the codon usage bias is strong), calculated by counting the ratio of the optimal codon number to the total synonymous codon number in one specific gene (Li et al., 2023). The ENC considers the number of synonymous codons used for each amino acid and the frequency with which they are used in the sequence. And the ENC value ranges from 20 to 61, which is lower than 35, the codon usage bias is strong; while if it’s higher than 35, the codon is randomly used (Tyagi and Nagar, 2022). The general average hydropathicity (GRAVY) values range from −2 to 2; positive and negative values represent hydrophobic and hydrophilic proteins, respectively (Munjal et al., 2020). The aromaticity (AROMO) value represents the frequency of aromatic amino acids (Phe, Tyr, and Trp) in a specific gene (Khandia et al., 2019). Besides, the length of synonymous codons (L_sym) and length of amino acids (L_aa), the two indices represent the number of synonymous codons and the number of translatable codons, respectively (Yang et al., 2021).

### Analysis of relative synonymous codon usage

Relative synonymous codon usage (RSCU) value is an index used to analyze the codon usage bias in genomic or transcriptomic sequences. It compares the frequency of each synonymous codon (a codon that codes for the same amino acid) to the expected frequency if all synonymous codons for that amino acid were used equally. RSCU values range from 0.5 to 2.0 and can reveal information about evolutionary conserved regions, gene expression, and nucleotide composition biases in a genome or transcriptome. A RSCU value >1 indicates a positive codon bias (RSCU value >1.6 indicates a strong positive codon bias), while an RSCU value <1 indicates a negative codon bias, and an RSCU value =1 indicates a random codon usage (Beelagi et al., 2021).

### Neutrality plot analysis

The neutrality plot can explain the balance between mutation pressure and natural selection in specific genes. The line of regression slope between GC3 and GC12 (the average GC codon content in GC1 and GC2) indicates that mutation pressure is the major factor affecting CUB when values come close to 1. In contrast, if there is no correlation between GC12 and GC3, the value comes close to 0, then the main driving force of the tested gene is natural selection (Patil et al., 2021).

### PR2-bias plot analysis

Parity Rule 2 bias (PR2-Bias) plot analysis were performed based on [A3/(A3 + U3) vs. G3/(G3 + C3)]. If the codon has no usage bias, A = T and C = G, the value was in the center point of the plot. In contrast, the other vectors emitted from the center point indicate the degree and direction of the gene bias (Huang et al., 2022).

### ENC-GC3 plot analysis

The ENC-GC3 plot (ENC vs. GC3) is usually used to analyze the influencing factor of CUB in a specific gene, and the standard curve shows the functional relation between ENC and GC3. If the corresponding points are distributed around or on the standard curve, we can conclude that the mutation pressure is an independent force in CUB. In contrast, the natural selection factor may play a key role in the formation of codon bias (Kumar et al., 2021).

### Correlation analysis

Correlation analysis was performed to explain the relationship among codon base composition (GC1, GC2, GC3, GCs), CAI, CBI, FOP, ENC, GRAVY, AROMO, L_sym, and L_aa of PHIST proteins. Pearson correlation analysis method was applied in correlation analysis. All processes were executed using the R corrplot package (Liu H. et al., 2020).

### Phylogenetic analysis

The evolutionary relationships of 88 PHIST proteins were analyzed by RSCU results and amino acid sequences, respectively. The phylogenetic tree was constructed using the neighbor-joining method by MEGA 11.0^2^ and a cluster heat map was generated by Hemi 1.0 software.^3^

### Software used

All indices of CUB were calculated by CodonW software 1.4.2.^4^ Clustering and correlation analyses were conducted using the statistical software SPSS 18.0, and statistical significance was defined by a value of *p* < 0.05. Graphs were generated in GraphPad Prism 6.01.^5^

## Results

### Results of codon base composition in PHIST proteins

**Figure 1:**
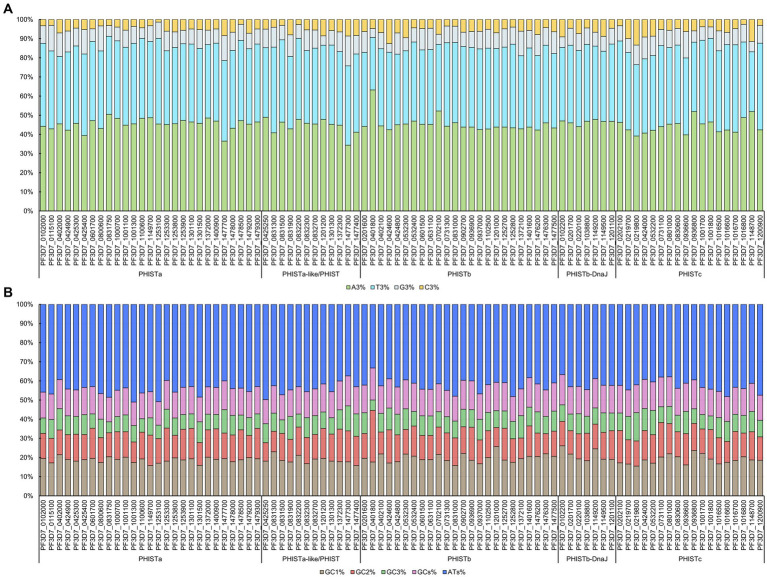
Codon base composition in PHIST proteins. The codon base composition of PHIST proteins was determined. **(A)** The percentages of nucleotides at the third codon location (C3%, T3%, G3%, and A3%) in PHIST proteins. **(B)** The GC% content at all three codon locations (GC1%, GC2%, and GC3%) and the total GCs% and ATs% content in PHIST proteins. The X-axis represents different PHIST protein subgroups, while the Y-axis represents relative percentages.

CUB can be significantly influenced by the general base composition of genomes. We chose 88 PHIST proteins from *Plasmodium falciparum* for analysis of codon usage (Supplementary Table S1). Our statistical analysis showed that the length of the encoding region for these PHIST proteins ranged from 255 bp to 3,657 bp, with PF3D7_0801000 having the longest length with a PRESAN (*Plasmodium* Ring-infected erythrocyte surface antigen N-terminal) domain and PF3D7_1149700 having the shortest length without any conserved domain (Table 1). We further calculated the base composition of 88 PHIST proteins and our results showed that all the 88 PHIST proteins are rich in the A3, T3, and ATs bases (Figure 1 and Supplementary Table S1). The content of A3% is most in PF3D7_0401800 (77.08%) and least in PF3D7_1477300 (46.49%), and the T3% content of PF3D7_0831300 (67.81%) is at a maximum level higher than that in others. Though the content of C3% (21.08%) in PF3D7_1477300 and G3% (18.97%) in PF3D7_0219800 are two most among these proteins, the content is still much lower than A3/T3% (Figure 1A and Supplementary Table S1). In addition, analysis of nucleotide content at different synonymous codon positions showed that the values of GC2% ranged from 15.38 to 54.01% (mean: 24.52%), while GC3% ranged from 9.34 to 26.25% (mean: 16.43%). However, the GC1% values ranged from 25.89 to 49.72%, with the average value (33.60%) being higher than GC2% and GC3%. Besides, the content of ATs% (ranged from 66.61 to 80.65%, mean: 75.15%) is two to three times that of GCs% (Figure 1B and Supplementary Table S1).

### Results of codon usage index analysis in PHIST proteins

We conducted an analysis on 88 PHIST proteins belonging to 5 subgroups and determined their CAI values. The range of CAI values for these proteins was 0.125 to 0.274, as shown in Table 1. Notably, the gene PF3D7_1201000 exhibited the highest CAI value, while PF3D7_1478500 had the lowest CAI value, suggesting a strong codon bias for PF3D7_1201000. When considering the different subgroups, the PHISTb-DnaJ subgroup showed the highest average CAI value of 0.183, followed by the PHISTb subgroup with a value of 0.180. On the other hand, the PHISTa-like/PHIST subgroup had the lowest mean CAI value of 0.159, which was similar to that of the PHISTa subgroup (0.160), indicating relatively weaker codon bias for both PHISTa-like/PHIST and PHISTa subgroups. The 88 PHIST proteins that were analyzed showed a range of CBI values from −0.412 to −0.033 (Table 1). Notably, PF3D7_0401800 had the lowest CBI value, while PF3D7_0102200 had the highest CBI value, indicating a strong codon bias for PF3D7_0102200. Among the 88 PHIST proteins, the average FOP values ranged from 0.225 to 0.416 (Table 1). PF3D7_1149700 had the lowest FOP value, while PF3D7_0102200 had the highest FOP value with strong codon bias. The GRAVY values were calculated for the 88 PHIST proteins, and 87 of them had negative values, suggesting that they are likely hydrophilic proteins (Table 1). However, PF3D7_1477400 from the PHISTa-like/PHIST subgroup was considered hydrophobic. The aromatic amino acid (AROMO) values ranged from 0.061 to 0.176 (Table 1). PF3D7_1149700 had the highest AROMO value, while PF3D7_0402000 had the lowest AROMO value. The AROMO values varied significantly among different PHIST proteins, with an average of 0.111. The average ENC value for the 88 PHIST proteins ranged from 29.78 (PF3D7_1016600) to 49.72 (PF3D7_0219800), with an average ENC value of 36.69. Among the 88 proteins, 26 had ENC values below 35, with a significant portion belonging to the PHISTa subgroup. The remaining 62 proteins had ENC values above 35, indicating a weaker codon usage preference (Table 1). Additionally, Table 1 provides data on L_sym (ranging from 80 to 1,182) and L_aa (ranging from 85 to 1,219).

### Defining codon usage patterns in PHIST proteins

**Figure 2:**
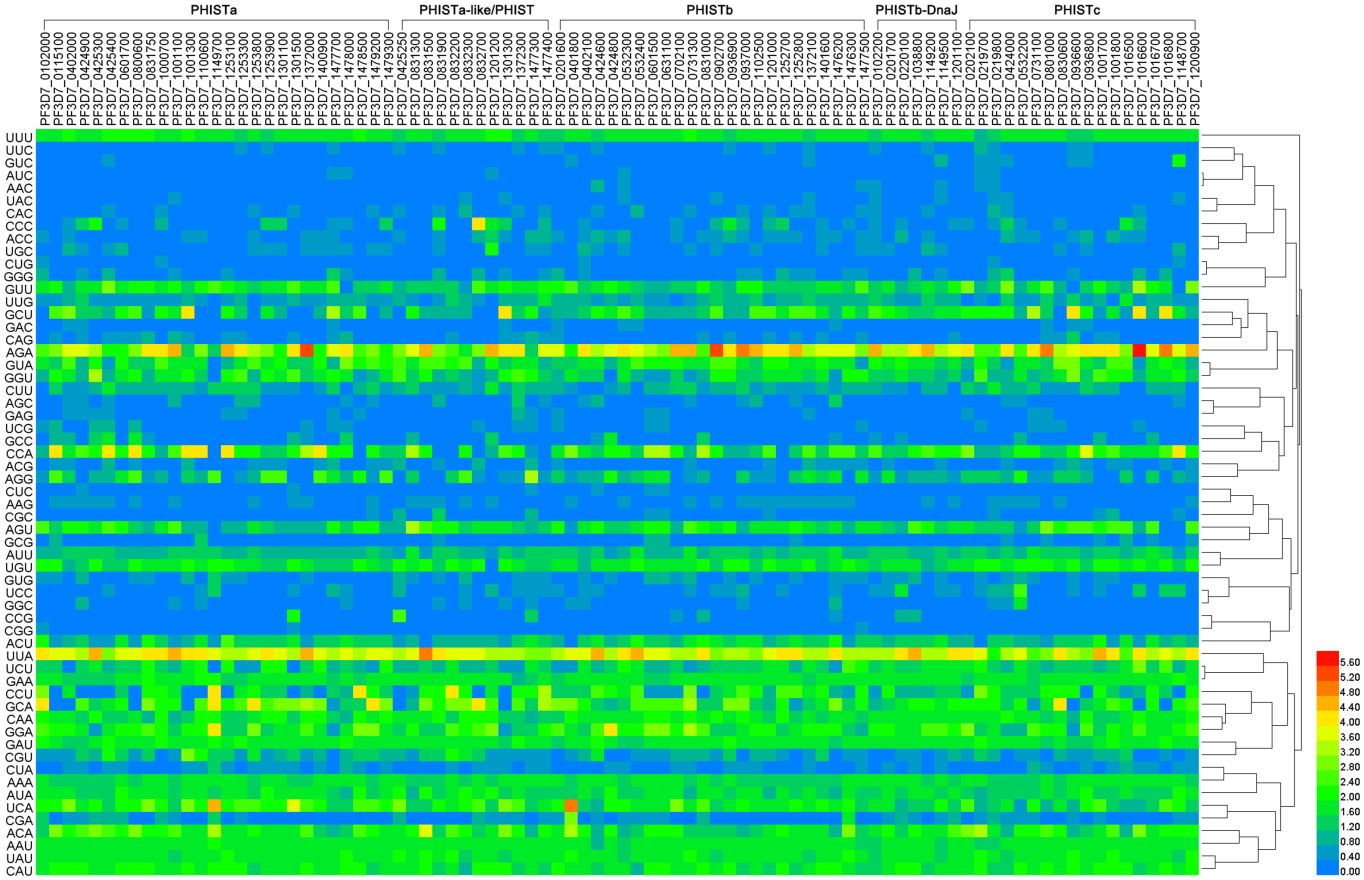
The RSCU values in PHIST proteins. The relative synonymous codon usage (RSCU) value was calculated by dividing the number of amino acids encoded by the same codon by their frequency of appearance in the same codon. The color of the color block changes from blue to red, indicating that the RSCU values are increasing, with an RSCU value >1 indicating positive codon bias. The homology of codons is shown on the right side and the subgroups are shown on the top side of the figure.

The PHIST proteins were analyzed using an RSCU analysis to regulate the same pattern of codon usage. It was observed that CUB occurs in these proteins, and 87 out of the 88 PHIST proteins exhibited more than 22 positive codon biases (RSCU≥1), except for PF3D7_1301300, which had 21 positive codon biases (Figure 2 and Supplementary Table S2). Additionally, among the 88 PHIST proteins, more than 11 high-frequency codons (RSCU≥1.6) were found in the PHISTa subgroup (PF3D7_0601700, PF3D7_1000700, and PF3D7_1100600) and PHISTc subgroup (PF3D7_1016500 and PF3D7_1016700), indicating a stronger positive codon bias. Only 11 high-frequency codons were present in PF3D7_0424000. Furthermore, the RSCU analysis revealed that the most frequently used codons in the 88 PHIST proteins are AGA (Arg) and UUA (Leu), whereas CGG (Arg) is rarely used, and is even absent in the PHISTa-like/PHIST and PHISTb subgroups. Among the optimal codons, PF3D7_1016600 has the highest value with AGA (Arg, RSCU = 6), followed by PF3D7_0401800 with UCA (Ser, RSCU = 4.85) and PF3D7_0831500 with UUA (Leu, RSCU = 4.85), indicating the strongest positive codon bias. On the other hand, PF3D7_0532400 has the lowest value with AAC (Asn, RSCU = 0.03) among the 59 synonymous codons. Furthermore, AGA (Arg) serves as the optimal codon in the PHISTa, PHISTb, PHISTb-DnaJ, and PHISTc subgroups, whereas UUA (Leu) is the optimal codon in the PHISTa-like/PHIST subgroup.

### Results of neutrality plot analysis in PHIST proteins

**Figure 3:**
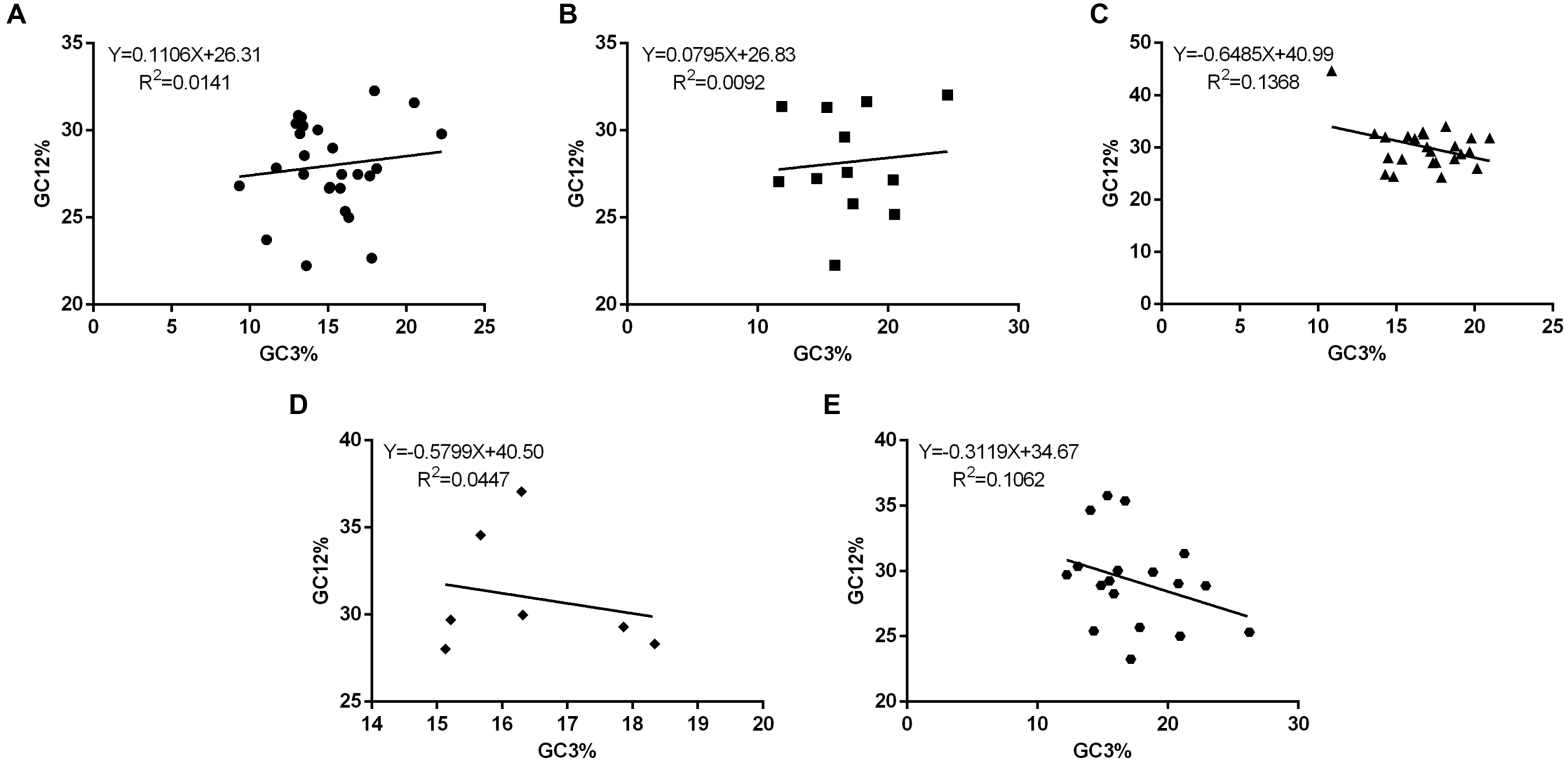
The neutrality plot analysis in PHIST proteins. The correlations between the GC12 and GC3 in PHIST proteins were analyzed, while calculating the standard curve and *R*^2^, respectively. The X-axis represents GC3%, while the Y-axis represents GC12%. **(A)** PHISTa subgroup. **(B)** PHISTa-like/PHIST subgroup. **(C)** PHISTb subgroup. **(D)** PHISTb-DnaJ subgroup. **(E)** PHISTc subgroup.

To determine the impact of mutation pressure and natural selection on CUB in the 88 PHIST proteins, a plot of neutrality was generated to analyze the relationship between GC12 and GC3 composition. The GC12 content ranged from 22.23 to 44.65%, while the GC3 content varied from 9.34 to 26.25% (Supplementary Table S1). To examine this association further, we plotted the neutrality paradigm for the 88 PHIST proteins, which were categorized into five subgroups: A. PHISTa subgroup. B. PHISTa-like/PHIST subgroup. C. PHISTb subgroup. D. PHISTb-DnaJ subgroup. E. PHISTc subgroup. The regression lines’ slopes ranged from −0.6485 (PHISTb subgroup) to 0.1106 (PHISTa subgroup), indicating a weak association between GC12 and GC3 content in the PHIST proteins (Figure 3). Furthermore, the *R*^2^ values of the standard curve varied from 0.0092 (PHISTa-like/PHIST subgroup) to 0.1368 (PHISTb subgroup). The statistical analysis showed no significant correlation (*p* > 0.05) between the GC12 and GC3 values, suggesting that natural selection might be a driving force in the evolution of PHIST proteins in *Plasmodium falciparum*, consistent with previous studies.

### Results of PR2-bias plot analysis in PHIST proteins

**Figure 4:**
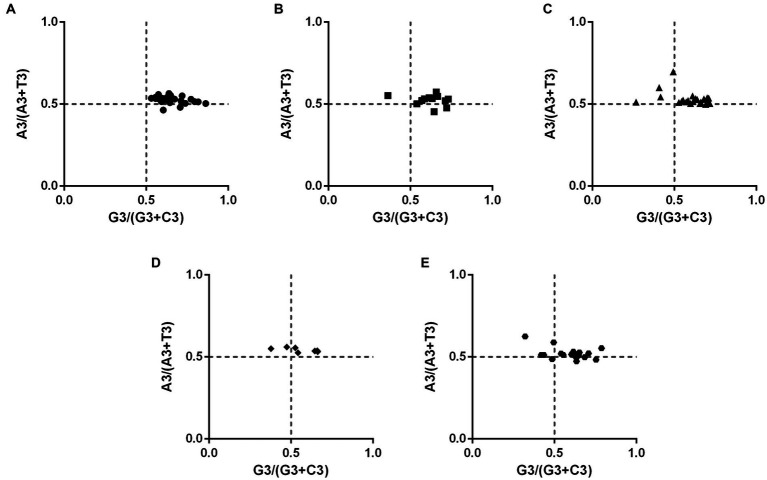
The PR2-bias plot analysis in PHIST proteins. The correlations between A3/(A3 + U3) and G3/(G3 + C3) were analyzed in PHIST proteins, respectively. If a codon has no usage bias, the value will be in the center point of the plot. The first quadrant represents the codon preference for A/G, while the third quadrant represents the preference for T/C. The X-axis represents GC3%, while the Y-axis represents GC12%. **(A)** PHISTa subgroup. **(B)** PHISTa-like/PHIST subgroup. **(C)** PHISTb subgroup. **(D)** PHISTb-DnaJ subgroup. **(E)** PHISTc subgroup.

To investigate potential biases in PHIST proteins of *Plasmodium falciparum*, we conducted a Parity Rule 2 (PR2) plot analysis (Figure 4). The plot was divided into four quadrants with both axes centered on 0.5. In the first quadrant (upper right), A and G were identified as the optimal codons, while the third quadrant favored T and C. The majority of PHIST proteins were found in the first quadrant, with a minority exhibiting a preference for A over T and C over G (located in the second quadrant). Very few proteins were observed in the third quadrant. These findings indicate that factors other than codon bias, such as natural selection, play a significant role in shaping the codon usage patterns of PHIST proteins in *Plasmodium falciparum*.

### Results of ENC-GC3 plot analysis in PHIST proteins

**Figure 5:**
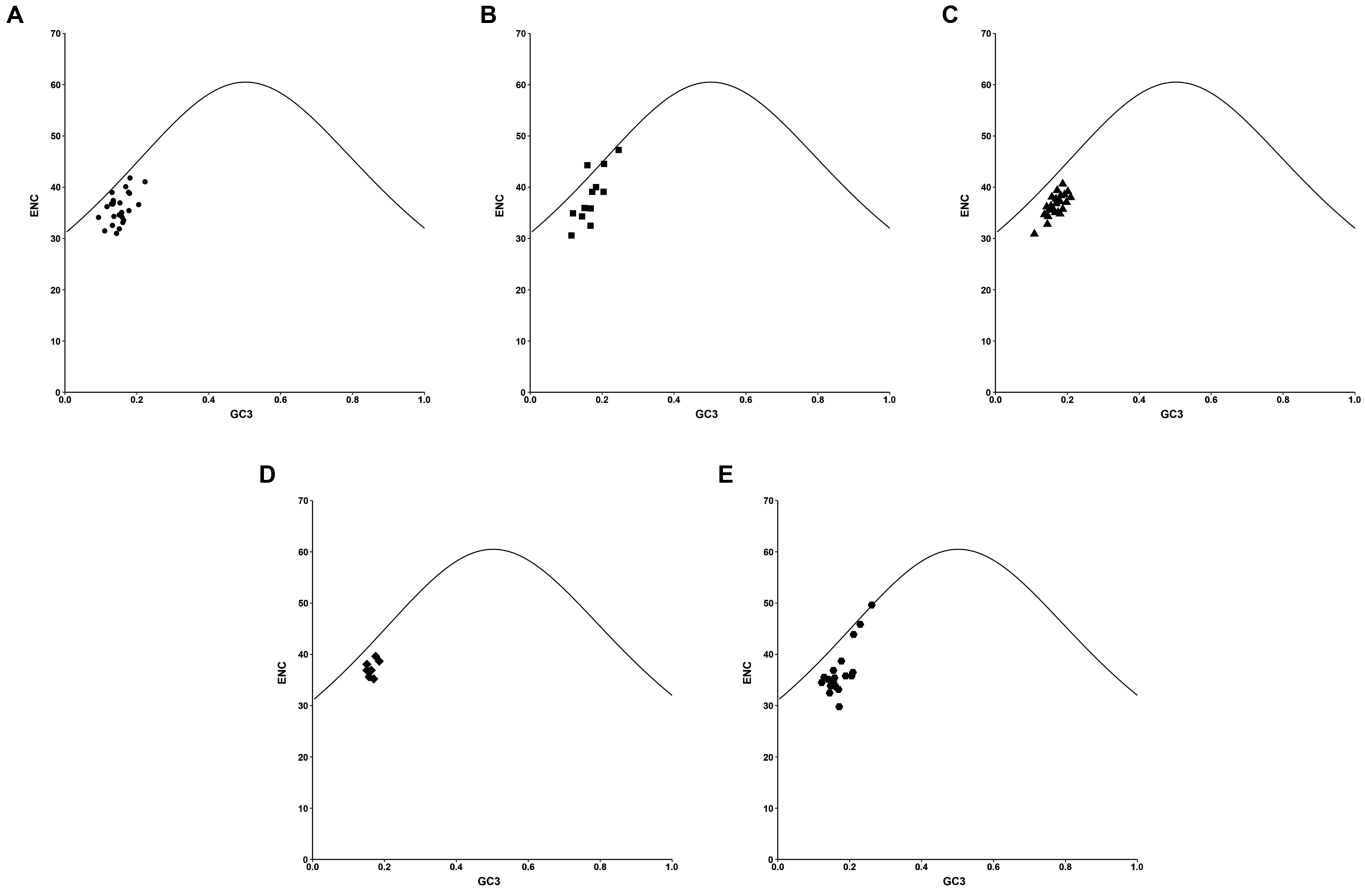
The ENC-GC3 plot analysis in PHIST proteins. The correlations between the effective number of codons (ENC) and the nucleotide G/C content at the third codon location (GC3) were analyzed in PHIST proteins, respectively. The standard curve represents the functional relationship between ENC and GC3 under mutation pressure rather than natural selection. The X-axis represents GC3%, while the Y-axis represents the ENC value. **(A)** PHISTa subgroup. **(B)** PHISTa-like/PHIST subgroup. **(C)** PHISTb subgroup. **(D)** PHISTb-DnaJ subgroup. **(E)** PHISTc subgroup.

To validate the impact of GC3s on the codon bias of PHIST proteins in *Plasmodium falciparum*, we utilized a distribution plot with varying usage of codons (Figure 5). ENC values were compared to the corresponding GC3 values, and the resulting standard curve revealed that the relationship between ENC and GC3 is primarily influenced by mutation pressure rather than natural selection. In cases where the gene’s GC content reflects mutational pressure, all plot points align with the expected curve, indicating random codon usage. Conversely, if natural selection exerts pressure on the gene, most plot points deviate below the expected curve, with only one point (PF3D7_0425250) surpassing it. Our findings demonstrate that while mutation pressure may contribute to codon bias, natural selection also plays a pivotal role, as indicated by the majority of plot points closely aligning with the standard curve, with only one point (PF3D7_0219800) falling directly on it.

### Results of correlation analysis in PHIST proteins

**Figure 6:**
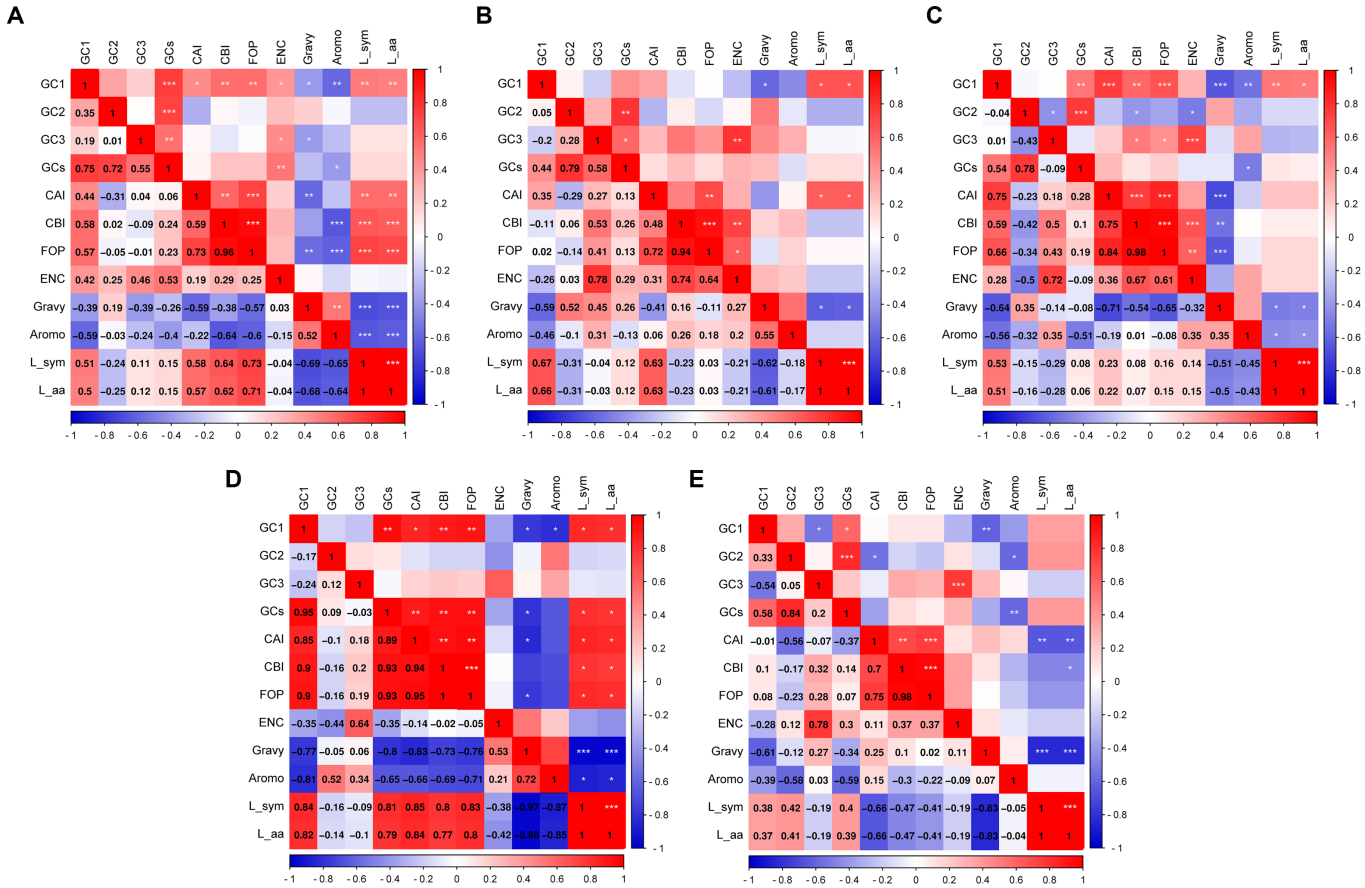
The correlation analysis in PHIST proteins. The correlations among codon base composition (GC1, GC2, GC3, GCs), codon adaptation index (CAI), codon bias index (CBI), frequency of optimal codons (FOP), the effective number of codons (ENC), general average hydropathicity (GRAVY), aromaticity (AROMO), length of synonymous codons (L_sym) and length of amino acids (L_aa) were analyzed in PHIST proteins, respectively. The color of the color block changes from blue to red, indicating that the correlation is increasing. The scales on the right and bottom represent the strength of the correlation, while the components on the left and top represent different indices. **(A)** PHISTa subgroup. **(B)** PHISTa-like/PHIST subgroup. **(C)** PHISTb subgroup. **(D)** PHISTb-DnaJ subgroup. **(E)** PHISTc subgroup. One asterisk (*) indicates a significant correlation among indices at the *p* < 0.05; Two asterisks (**) indicate the correlation at the *p* < 0.01; Three asterisks (***) indicate the correlation at the *p* < 0.001.

To visually present the indices associated with the 12 primary contributors, we calculated correlations among the crucial indices to identify the key factors contributing to codon bias (Figure 6). In the PHISTa subgroup, the GC2 value exhibited a significant correlation only with GCs (*p* < 0.001), whereas GC1 showed correlations with nearly all the indices (Figure 6A). Conversely, in the PHISTb-DnaJ subgroup, neither the GC2 nor GC3 values demonstrated correlations with other indices (Figure 6D). Additionally, except for the PHISTc subgroup, we did not observe a significant correlation between GC1 and GC2 or GC3 in the PHISTa, PHISTa-like/PHIST, PHISTb, and PHISTb-DnaJ subgroups (Figure 6). On the other hand, FOP values showed a significant correlation with the CBI among these PHIST proteins (*p* < 0.001), and there was a notable correlation between ENC and GC3 contents in the PHISTa, PHISTa-like/PHIST, PHISTb, and PHISTc subgroups, suggesting a possible influence of natural selection on the usage of synonymous codons. Moreover, only a few indices showed correlations in the PHISTa-like/PHIST subgroup (Figure 6B), whereas nearly all indices correlated in the PHISTa and PHISTb subgroups (Figures 6A,C), indicating that both mutation pressure and natural selection play key roles in the formation of codon bias.

### Results of phylogenetic analysis in PHIST proteins

**Figure 7:**
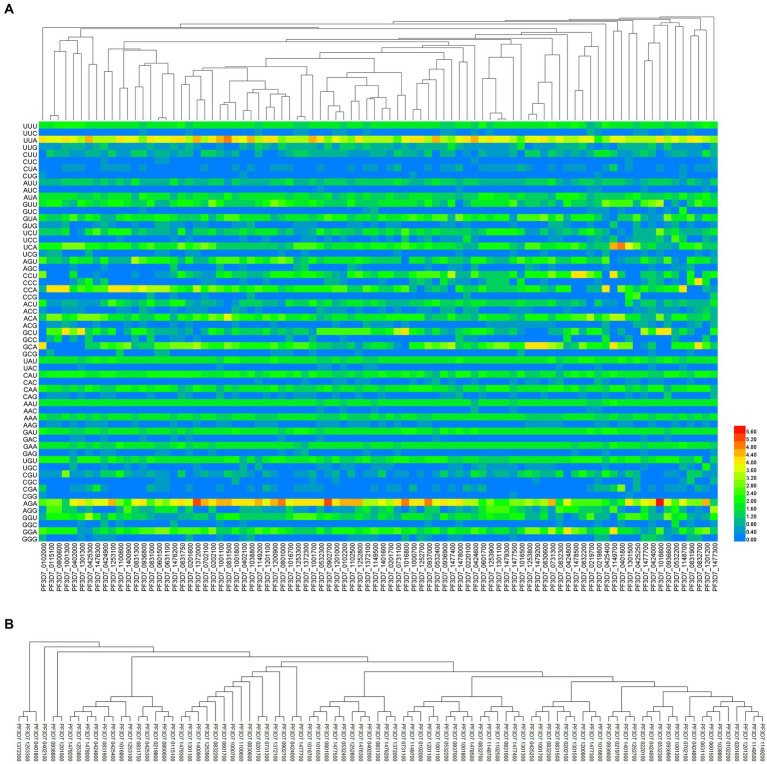
The phylogenetic analysis in PHIST proteins. The evolution analysis among 88 PHIST proteins was clustered by relative synonymous codon usage (RSCU) value **(A)**, the color of the color block changes from blue to red, indicating that the RSCU values are increasing and coding sequences **(B)**, by the neighbor-joining method, respectively.

To evaluate how evolutionary processes affect the codon usage pattern of PHIST proteins in *Plasmodium falciparum*, we employed RSCU values of 88 PHIST proteins for cluster analysis (Figure 7A). The findings revealed that the proteins were categorized into multiple clusters based on evolutionary distance. Most PHIST proteins in the PHISTa and PHISTb subgroups were assigned to separate clusters, whereas the PHISTa-like/PHIST, PHISTb-DnaJ, and PHISTc subgroups occupied distinct clusters. Unexpectedly, PF3D7_0831750 and PF3D7_1372000 of the PHISTa subgroup were placed in the same cluster as PF3D7_0201600 and PF3D7_0702100 of the PHISTb subgroup. To compare with the RSCU-based phylogenetic relationship, a neighbor-joining method was utilized for phylogenetic analysis based on CDS (Figure 7B). According to the CDS phylogenetic analysis, PF3D7_0831750 of the PHISTa subgroup exhibited a different evolutionary clade within the same cluster as PF3D7_0425300 and PF3D7_1253100 of the PHISTa subgroup, indicating a closer evolutionary relationship.

## Discussion

Over the course of long-term evolution, organisms will eventually develop a specific set of codon usages that maintain the transmission of genetic information between nucleotides and amino acids (Hershberg and Petrov, 2008; Chakraborty et al., 2020). However, different genes within the same species or across different species exhibit preferences for codon usage (Liu X. Y. et al., 2020). As a result, the analysis of CUB provides valuable insights into the regulatory mechanisms of translation processes and enables the prediction and optimization of exogenous genes for enhanced expression levels through industrial modification, even helping identify key functional sites (Yu et al., 2021; Wu et al., 2022). Currently, a complete understanding of codon usage characteristics for PHIST proteins in *Plasmodium falciparum* is lacking. *Plasmodium falciparum* is a protozoan parasite responsible for causing the most severe type of malaria in humans. Artemisinin has a good treatment effect on this disease, however, in some areas, such as Rwanda and Uganda, *Plasmodium falciparum* have developed partial resistance to artemisinin and even artemisinin-based combination drugs, resulting in the appearing of artemisinin-resistant Kelch13 mutant strains of *Plasmodium falciparum* (Balikagala et al., 2021; Dhorda et al., 2021). Therefore, the development of new antimalarial drugs or vaccines is urgently needed. Therefore, it is necessary to search for new drug targets. In previous studies, a protein (PF3D7_1372300) in the PHIST family was found to interact with PfEMP1, thus PHIST proteins may become potential new targets (Yang et al., 2020). PHIST proteins have been found to interact with host proteins also, such as cytoskeletal components and immune signaling molecules, to aid in the invasion and survival of the parasite within the host organism (Warncke et al., 2020; Shakya et al., 2022; Tripathi et al., 2022). Understanding the function and role of these PHIST proteins is crucial for developing effective strategies against malaria.

Research on PHIST proteins has revealed their diverse functions and involvement in various stages of the *Plasmodium* life cycle. PHIST proteins in different subgroups display significant variations in terms of their length and codon base composition. The average amino acid length of PHISTa subgroup is shorter than other subgroups, among which the length of PF3D7_1149700 is shortest. Though PHISTb-DnaJ subgroup possess the longest average amino acid length, PF3D7_0801000 of PHISTc subgroup is the longest among these PHIST proteins, indicating differentiation within the *Plasmodium falciparum* species (Table 1). Notably, the differences in synonymous codons primarily lie in the third codon position. Interestingly, our study found that all 88 identified PHIST proteins tend to end with A/T, which is consistent with previous research on *Mycoplasma capricolum* and *Onchocerca volvulus*, where enrichment of A and T at the end of genes was observed (Mazumder et al., 2018). The average values of A% and T% in the five subgroups were 62.50 to 65.06% and 54.90 to 58.13%, respectively, while the maximum average value of G/C% is only 13.47% of the PHISTa-like/PHIST subgroup. The T3% content of PF3D7_0401800 (33.48%) in PHISTb subgroup is the lowest among these proteins, but it is still much higher than that of G3% of PF3D7_1477300 (21.08%) from PHISTa-like/PHIST subgroup (Figure 1 and Supplementary Table S1), which proved that one specific gene shows diverse codon usage bias in the same or different species and the results are consistent with the feature of apicomplexan protozoa codon usage in other genes (Lamolle et al., 2022; Benisty et al., 2023). Most high-frequency PHIST protein codons are AGA (Arg) and UUA (Leu) analyzed by RSCU, indicating the strongest positive codon bias. However, CGG (Arg) is seldom used and has never appeared in the codon encoding the PHISTa-like/PHIST and PHISTb subgroups proteins, which also show the same tendency of using the third codon in *Plasmodium falciparum* (Figure 2 and Supplementary Table S2).

The values of CAI, CBI, FOP, and ENC are also analyzed in this paper. The CAI value of PF3D7_1201000 (0.274) in PHISTb subgroup is highest and the average CAI value of PHISTb-DnaJ subgroup (0.183) is higher than others, which indicates a strong codon bias in PF3D7_1201000 and PHISTb-DnaJ subgroup. Meanwhile, the average CAI value of the PHISTa-like/PHIST subgroup (0.159) and PHISTa subgroup (0.160) is almost the same, with a weak codon bias (Table 1). Additionally, specific PHIST genes, such as PF3D7_0102200 in the PHISTb-DnaJ subgroup, show a strong bias towards certain codons, as indicated by higher values of CBI and FOP. However, the average values of CBI and FOP in PHISTa-like/PHIST subgroup are the lowest, indicating the random codon usage within the subgroup. Besides, an ENC value below 35 signifies a strong preference for certain codons, an observation supported by some of the ENC values in our study (Prabha et al., 2017; Pepe and Keersmaecker, 2020). Overall, the average ENC value of the 88 PHIST proteins is 36.69, though 26 out of 88 ENC values were lower than 35, indicating a weak codon preference in *Plasmodium falciparum* PHIST proteins. Interestingly, the PHISTa subgroup has the lowest average ENC value (36.03) while PHISTa-like/PHIST subgroup is highest (38.19), suggesting a stronger codon preference of PHISTa subgroup compared to other subgroups (Table 1). Furthermore, our analysis revealed significant correlations among codon base composition (GC1, GC2, GC3, GCs), CAI, CBI, FOP, ENC, GRAVY, AROMO, L_sym, and L_aa. And these correlations indicate the influence of base composition and codon usage indices on CUB, particularly with respect to GC1 (Figure 6). Mutation pressure and natural selection are two main factors affecting CUB, and the neutrality plot analysis, PR2-bias plot analysis, and ENC-GC3 plot analysis further support the role of natural selection in shaping the codon bias of PHIST proteins in *Plasmodium falciparum* (Figures 3–5). Although there are some differences in codon usage indices among the five PHIST protein subgroups, it is evident that the CUB of these proteins is influenced by strong natural selection.

Currently, RSCU clustering and CDS phylogenetic tree are commonly used for analyzing the evolutionary relationship of genes within the same or different species (Jiang et al., 2023). These two clustering analysis methods yield consistent results in certain subgroups, but their results may significantly differ in others. In this study, we examined the relationship among PHIST proteins in different subgroups of *Plasmodium falciparum* based on CDS and RSCU analyses, respectively. Interestingly, the phylogenetic relationships based on CDS analysis were deemed to be more reliable compared to those based on RSCU analysis. In the RSCU clustering analysis, the unexpected grouping of PF3D7_0831300 (PHISTa-like/PHIST subgroup), PF3D7_0936800 (PHISTc subgroup), and PF3D7_0831000 (PHISTb subgroup) occurred within the same cluster. However, the genetic relationships among some subgroups were accurately interpreted based on the RSCU values, which were consistent with those obtained from the CDS phylogenetic tree. For example, the relationships between PF3D7_0115100 and PF3D7_0800600 in the PHISTa subgroup, PF3D7_0601500 and PF3D7_0631100 in the PHISTb subgroup, and PF3D7_0801000, PF3D7_1016700, and PF3D7_1200900 in the PHISTc subgroup were correctly identified. These results indicate that the phylogenetic outcomes based on RSCU analysis can serve as valuable supplementary information to those derived from sequence-based methods (Figure 7).

## Conclusion

In summary, the codon usage patterns of PHIST proteins in *Plasmodium falciparum* are influenced by various factors, with natural selection being the primary driving force and mutation pressure playing a relatively minor role. Exploring these proteins as targets could open up new possibilities for the development of antimalarial drugs or vaccines. Nevertheless, additional research is required to comprehensively understand the exact mechanisms of PHIST proteins and their potential as therapeutic targets.

## Data availability statement

The original contributions presented in the study are included in the article/Supplementary material, further inquiries can be directed to the corresponding author.

## Author contributions

BY: Conceptualization, Funding acquisition, Methodology, Resources, Software, Supervision, Validation, Writing – original draft, Writing – review & editing. ZC: Methodology, Software, Validation, Writing – original draft, Resources. LL: Methodology, Writing – original draft, Software, Validation. KC: Methodology, Software, Validation, Writing – original draft. SG: Methodology, Software, Validation, Writing – original draft. YS: Methodology, Software, Validation, Writing – original draft. CL: Methodology, Software, Validation, Writing – original draft. DW: Conceptualization, Funding acquisition, Investigation, Methodology, Project administration, Resources, Software, Supervision, Validation, Writing – original draft, Writing – review & editing.

## Footnotes

^1^ https://plasmodb.org/plasmo/app

^2^ https://www.megasoftware.net/

^3^ http://hemi.biocuckoo.org/down.php

^4^ http://codonw.sourceforge.net/

^5^ http://www.graphpad.com/scientific-software/prism/

## References

[ref1] Alqahtani T. Khandia R. Puranik N. Alqahtani A. M. Chidambaram K. Kamal M. A. (2022). Codon usage is influenced by compositional constraints in genes associated with dementia. Front. Genet. 13:884348. doi: 10.3389/fgene.2022.884348 36017501 PMC9395603

[ref2] Bailey S. F. Alonso Morales L. A. Kassen R. (2021). Effects of synonymous mutations beyond codon bias: the evidence for adaptive synonymous substitutions from microbial evolution experiments. Genome Biol. Evol. 13:evab141. doi: 10.1093/gbe/evab141 34132772 PMC8410137

[ref3] Balikagala B. Fukuda N. Ikeda M. Katuro O. T. Tachibana S. I. Yamauchi M.. (2021). Evidence of artemisinin-resistant malaria in Africa. N. Engl. J. Med. 385, 1163–1171. doi: 10.1056/NEJMoa2101746 34551228

[ref4] Beelagi M. S. Kumar S. S. Indrabalan U. B. Patil S. S. Prasad A. Suresh K. P.. (2021). Synonymous codon usage pattern among the S, M, and L segments in Crimean-Congo hemorrhagic fever virus. Bioinformation 17, 479–491. doi: 10.6026/97320630017479 34602775 PMC8450151

[ref5] Benisty H. Hernandez-Alias X. Weber M. Anglada-Girotto M. Mantica F. Radusky L.. (2023). Genes enriched in a/T-ending codons are co-regulated and conserved across mammals. Cell Syst. 14, 312–323.e3. doi: 10.1016/j.cels.2023.02.002 36889307

[ref9001] Bhattacharyya D. Uddin A. Das S. Chakraborty S. (2019). Mutation pressure and natural selection on codon usage in chloroplast genes of two species in Pisum L. (Fabaceae: Faboideae). Mitochondrial. DNA. A. DNA. Mapp. Seq. Anal. 30:664–673. doi: 10.1080/24701394.2019.1616701 31119964

[ref6] Boissinot S. (2022). On the base composition of transposable elements. Int. J. Mol. Sci. 23:4755. doi: 10.3390/ijms23094755 35563146 PMC9099904

[ref7] Chakraborty S. Yengkhom S. Uddin A. (2020). Analysis of codon usage bias of chloroplast genes in Oryza species: codon usage of chloroplast genes in Oryza species. Planta 252:67. doi: 10.1007/s00425-020-03470-7 32989601

[ref8] Chaney J. L. Clark P. L. (2015). Roles for synonymous codon usage in protein biogenesis. Annu. Rev. Biophys. 44, 143–166. doi: 10.1146/annurev-biophys-060414-034333 25747594

[ref9] Chassalevris T. Chaintoutis S. C. Apostolidi E. D. Giadinis N. D. Vlemmas I. Brellou G. D.. (2020). A highly sensitive semi-nested real-time PCR utilizing oligospermine-conjugated degenerate primers for the detection of diverse strains of small ruminant lentiviruses. Mol. Cell. Probes 51:101528. doi: 10.1016/j.mcp.2020.101528 32004592

[ref10] Chen F. Yang J. R. (2022). Distinct codon usage bias evolutionary patterns between weakly and strongly virulent respiratory viruses. iScience 25:103682. doi: 10.1016/j.isci.2021.103682 34977494 PMC8704784

[ref11] Dhorda M. Amaratunga C. Dondorp A. M. (2021). Artemisinin and multidrug-resistant *plasmodium falciparum* – a threat for malaria control and elimination. Curr. Opin. Infect. Dis. 34, 432–439. doi: 10.1097/QCO.0000000000000766 34267045 PMC8452334

[ref12] Dilucca M. Pavlopoulou A. Georgakilas A. G. Giansanti A. (2020). Codon usage bias in radioresistant bacteria. Gene 742:144554. doi: 10.1016/j.gene.2020.144554 32173539

[ref13] Fierro M. A. Muheljic A. Sha J. Wohlschlegel J. A. Beck J. R. (2023). PEXEL is a proteolytic maturation site for both exported and non-exported *plasmodium* proteins. BioRxiv 2023:12.548774. doi: 10.1101/2023.07.12.548774 PMC10900883 38334391

[ref14] Gorlov I. P. Pikielny C. W. Frost H. R. Her S. C. Cole M. D. Strohbehn S. D.. (2018). Gene characteristics predicting missense, nonsense and frameshift mutations in tumor samples. BMC Bioinformatics 19:430. doi: 10.1186/s12859-018-2455-0 30453881 PMC6245819

[ref15] Hasan M. M. Polino A. J. Mukherjee S. Vaupel B. Goldberg D. E. (2023). The mature N-termini of *plasmodium* effector proteins confer specificity of export. MBio:e0121523. doi: 10.1128/mbio.01215-23 37646514 PMC10653839

[ref16] Hernandez-Alias X. Benisty H. Radusky L. G. Serrano L. Schaefer M. H. (2023). Using protein-per-mRNA differences among human tissues in codon optimization. Genome Biol. 24:34. doi: 10.1186/s13059-023-02868-2 36829202 PMC9951436

[ref17] Hershberg R. Petrov D. A. (2008). Selection on codon bias. Annu. Rev. Genet. 42, 287–299. doi: 10.1146/annurev.genet.42.110807.091442 18983258

[ref18] Hou W. (2020). Characterization of codon usage pattern in SARS-CoV-2. Virol. J. 17:138. doi: 10.1186/s12985-020-01395-x 32928234 PMC7487440

[ref19] Hu H. Dong B. Fan X. Wang M. Wang T. Liu Q. (2023). Mutational bias and natural selection driving the synonymous codon usage of single-exon genes in rice (*Oryza sativa* L.). Rice (N Y) 16:11. doi: 10.1186/s12284-023-00627-2 36849744 PMC9971424

[ref20] Huang X. Jiao Y. Guo J. Wang Y. Chu G. Wang M. (2022). Analysis of codon usage patterns in *Haloxylon ammodendron* based on genomic and transcriptomic data. Gene 845:146842. doi: 10.1016/j.gene.2022.146842 36038027

[ref21] Iriarte A. Lamolle G. Musto H. (2021). Codon usage bias: an endless tale. J. Mol. Evol. 89, 589–593. doi: 10.1007/s00239-021-10027-z 34383106

[ref22] Jiang Y. Neti S. S. Sitarik I. Pradhan P. To P. Xia Y.. (2023). How synonymous mutations alter enzyme structure and function over long timescales. Nat. Chem. 15, 308–318. doi: 10.1038/s41557-022-01091-z 36471044 PMC11267483

[ref23] Khandia R. Saeed M. Alharbi A. M. Ashraf G. M. Greig N. H. Kamal M. A. (2022). Codon usage bias correlates with gene length in neurodegeneration associated genes. Front. Neurosci. 16:895607. doi: 10.3389/fnins.2022.895607 35860292 PMC9289476

[ref24] Khandia R. Singhal S. Kumar U. Ansari A. Tiwari R. Dhama K.. (2019). Analysis of Nipah virus codon usage and adaptation to hosts. Front. Microbiol. 10:886. doi: 10.3389/fmicb.2019.00886 31156564 PMC6530375

[ref25] Kumar V. Behl A. Sharma R. Sharma A. Hora R. (2019). *Plasmodium* helical interspersed subtelomeric family-an enigmatic piece of the *plasmodium* biology puzzle. Parasitol. Res. 118, 2753–2766. doi: 10.1007/s00436-019-06420-9 31418110

[ref26] Kumar U. Khandia R. Singhal S. Puranik N. Tripathi M. Pateriya A. K.. (2021). Insight into codon utilization pattern of tumor suppressor gene EPB41L3 from different mammalian species indicates dominant role of selection force. Cancers (Basel) 13:2739. doi: 10.3390/cancers13112739 34205890 PMC8198080

[ref27] Lamolle G. Iriarte A. Musto H. (2022). Codon usage in the flatworm *Schistosoma mansoni* is shaped by the mutational bias towards a+T and translational selection, which increases GC-ending codons in highly expressed genes. Mol. Biochem. Parasitol. 247:111445. doi: 10.1016/j.molbiopara.2021.111445 34942292

[ref28] Lee S. K. Low L. M. Andersen J. F. Yeoh L. M. Valenzuela Leon P. C. Drew D. R.. (2023). The direct binding of *plasmodium vivax* AMA1 to erythrocytes defines a RON2-independent invasion pathway. Proc. Natl. Acad. Sci. U. S. A. 120:e2215003120. doi: 10.1073/pnas.2215003120 36577076 PMC9910450

[ref29] Leonard C. M. Uhomoibhi P. Abubakar A. Ogunniyi A. Mba N. Greby S. M.. (2023). Dynamics of IgG antibody response against *plasmodium* antigens among Nigerian infants and young children. Front. Immunol. 14:1208822. doi: 10.3389/fimmu.2023.1208822 37691957 PMC10484571

[ref30] Li Q. Luo Y. Sha A. Xiao W. Xiong Z. Chen X.. (2023). Analysis of synonymous codon usage patterns in mitochondrial genomes of nine *amanita* species. Front. Microbiol. 14:1134228. doi: 10.3389/fmicb.2023.1134228 36970689 PMC10030801

[ref31] Li G. Zhang L. Xue P. (2022). Codon usage divergence in Delta variants (B.1.617.2) of SARS-CoV-2. Infect. Genet. Evol. 97:105175. doi: 10.1016/j.meegid.2021.105175 34871776 PMC8641433

[ref32] Liu X. Y. Li Y. Ji K. K. Zhu J. Ling P. Zhou T.. (2020). Genome-wide codon usage pattern analysis reveals the correlation between codon usage bias and gene expression in *Cuscuta australis*. Genomics 112, 2695–2702. doi: 10.1016/j.ygeno.2020.03.002 32145379

[ref33] Liu H. Lu Y. Lan B. Xu J. (2020). Codon usage by chloroplast gene is bias in *Hemiptelea davidii*. J. Genet. 99:8. 32089527

[ref34] Masłowska-Górnicz A. van den Bosch M. R. M. Saccenti E. Suarez-Diez M. (2022). A large-scale analysis of codon usage bias in 4868 bacterial genomes shows association of codon adaptation index with GC content, protein functional domains and bacterial phenotypes. Biochim. Biophys. Acta Gene Regul. Mech. 1865:194826. doi: 10.1016/j.bbagrm.2022.194826 35605953

[ref35] Matsushita T. Kano-Sueoka T. (2023). Non-random codon usage of synonymous and non-synonymous mutations in the human HLA-A gene. J. Mol. Evol. 91, 169–191. doi: 10.1007/s00239-023-10093-5 36809491

[ref36] Mazumder G. A. Uddin A. Chakraborty S. (2018). Preference of a/T ending codons in mitochondrial ATP6 gene under phylum *Platyhelminthes*: codon usage of ATP6 gene in *Platyhelminthes*. Mol. Biochem. Parasitol. 225, 15–26. doi: 10.1016/j.molbiopara.2018.08.007 30149040

[ref37] Munjal A. Khandia R. Shende K. K. Das J. (2020). *Mycobacterium lepromatosis* genome exhibits unusually high CpG dinucleotide content and selection is key force in shaping codon usage. Infect. Genet. Evol. 84:104399. doi: 10.1016/j.meegid.2020.104399 32512206

[ref38] Mutisya J. M. Mobegi V. A. Kinyua J. K. Kivecu M. N. Okoth R. O. Chemwor G. C.. (2020). Characterization of sulfated polysaccharide activity against virulent *plasmodium falciparum* PHISTb/RLP1 protein. F1000Res 9:1268. doi: 10.12688/f1000research.26756.2 35600144 PMC9096147

[ref39] Pakrashi A. Patidar A. Singha D. Kumar V. Tyagi K. (2023). Comparative analysis of the two suborders of *Thysanoptera* and characterization of the complete mitochondrial genome of *Thrips parvispinus*. Arch. Insect Biochem. Physiol.:e22010. doi: 10.1002/arch.22010 36915951

[ref40] Parvathy S. T. Udayasuriyan V. Bhadana V. (2022). Codon usage bias. Mol. Biol. Rep. 49, 539–565. doi: 10.1007/s11033-021-06749-4 34822069 PMC8613526

[ref41] Patil S. S. Indrabalan U. B. Suresh K. P. Shome B. R. (2021). Analysis of codon usage bias of classical swine fever virus. Vet. World 14, 1450–1458. doi: 10.14202/vetworld.2021.1450-1458 34316191 PMC8304411

[ref42] Pepe D. De Keersmaecker K. (2020). Codon bias analyses on thyroid carcinoma genes. Minerva Endocrinol. 45, 295–305. doi: 10.23736/S0391-1977.20.03252-6 33103872

[ref43] Prabha R. Singh D. P. Sinha S. Ahmad K. Rai A. (2017). Genome-wide comparative analysis of codon usage bias and codon context patterns among cyanobacterial genomes. Mar. Genomics 32, 31–39. doi: 10.1016/j.margen.2016.10.001 27733306

[ref44] Pulido-Quevedo F. A. Arévalo-Pinzón G. Castañeda-Ramírez J. J. Barreto-Santamaría A. Patarroyo M. E. Patarroyo M. A. (2023). *Plasmodium falciparum* rhoptry neck protein 4 has conserved regions mediating interactions with receptors on human erythrocytes and hepatocyte membrane. Int. J. Med. Microbiol. 313:151579. doi: 10.1016/j.ijmm.2023.151579 37030083

[ref45] Shakya B. Kilili G. K. Wang L. Nakayasu E. S. LaCount D. J. (2022). Identification of exported *plasmodium falciparum* proteins that bind to the erythrocyte cytoskeleton. Microorganisms 10:1438. doi: 10.3390/microorganisms10071438 35889157 PMC9320996

[ref46] Shen G. Gao M. Cao Q. Li W. (2022). The molecular basis of FIX deficiency in hemophilia B. Int. J. Mol. Sci. 23:2762. doi: 10.3390/ijms23052762 35269902 PMC8911121

[ref47] Shen X. Song S. Li C. Zhang J. (2022). Synonymous mutations in representative yeast genes are mostly strongly non-neutral. Nature 606, 725–731. doi: 10.1038/s41586-022-04823-w 35676473 PMC9650438

[ref48] Tripathi J. Zhu L. Nayak S. Stoklasa M. Bozdech Z. (2022). Stochastic expression of invasion genes in *plasmodium falciparum* schizonts. Nat. Commun. 13:3004. doi: 10.1038/s41467-022-30605-z 35637187 PMC9151791

[ref49] Tyagi A. Nagar V. (2022). Genome dynamics, codon usage patterns and influencing factors in *Aeromonas hydrophila phages*. Virus Res. 320:198900. doi: 10.1016/j.virusres.2022.198900 36029927

[ref50] Vaz P. K. Armat M. Hartley C. A. Devlin J. M. (2023). Codon pair bias deoptimization of essential genes in infectious laryngotracheitis virus reduces protein expression. J. Gen. Virol. 104:001836. doi: 10.1099/jgv.0.001836 37010948

[ref51] Wang W. Blenner M. A. (2022). Engineering heterologous enzyme secretion in *Yarrowia lipolytica*. Microb. Cell Factories 21:134. doi: 10.1186/s12934-022-01863-9 PMC9252082 35786380

[ref52] Wang H. Liu S. Lv Y. Wei W. (2023). Codon usage bias of Venezuelan equine encephalitis virus and its host adaption. Virus Res. 328:199081. doi: 10.1016/j.virusres.2023.199081 36854361 PMC10194294

[ref53] Warncke J. D. Passecker A. Kipfer E. Brand F. Pérez-Martínez L. Proellochs N. I.. (2020). The PHIST protein GEXP02 targets the host cytoskeleton during sexual development of *plasmodium falciparum*. Cell. Microbiol. 22:e13123. doi: 10.1111/cmi.13123 31652487

[ref54] Warncke J. D. Vakonakis I. Beck H. P. (2016). *Plasmodium* helical interspersed subtelomeric (PHIST) proteins, at the center of host cell remodeling. Microbiol. Mol. Biol. Rev. 80, 905–927. doi: 10.1128/MMBR.00014-16 27582258 PMC5116875

[ref55] WHO. (2022). World malaria report 2022. Geneva: World Health Organization.

[ref56] Wiser M. F. (2023). Knobs, adhesion, and severe *falciparum* malaria. Trop. Med. Infect. Dis. 8:353. doi: 10.3390/tropicalmed8070353 37505649 PMC10385726

[ref57] Wu Y. Jin L. Li Y. Zhang D. Zhao Y. Chu Y.. (2021). The nucleotide usages significantly impact synonymous codon usage in *Mycoplasma hyorhinis*. J. Basic Microbiol. 61, 133–146. doi: 10.1002/jobm.202000592 33426673

[ref58] Wu C. Y. Xiao K. R. Wang L. Z. Wang J. Song Q. S. Stanley D.. (2022). Identification and expression profiling of serine protease-related genes in *Tenebrio molitor*. Arch. Insect Biochem. Physiol. 111:e21963. doi: 10.1002/arch.21963 36039637

[ref59] Yang B. Wang X. Jiang N. Sang X. Feng Y. Chen R.. (2020). Interaction analysis of a *plasmodium falciparum* PHISTa-like protein and PfEMP1 proteins. Front. Microbiol. 11:611190. doi: 10.3389/fmicb.2020.611190 33281807 PMC7691434

[ref60] Yang C. Zhao Q. Wang Y. Zhao J. Qiao L. Wu B.. (2021). Comparative analysis of genomic and transcriptome sequences reveals divergent patterns of codon bias in wheat and its ancestor species. Front. Genet. 12:732432. doi: 10.3389/fgene.2021.732432 34490050 PMC8417831

[ref61] Yu X. Liu J. Li H. Liu B. Zhao B. Ning Z. (2021). Comprehensive analysis of synonymous codon usage bias for complete genomes and E2 gene of atypical porcine *Pestivirus*. Biochem. Genet. 59, 799–812. doi: 10.1007/s10528-021-10037-y 33538926 PMC7860996

[ref62] Zhao Z. Y. Yu D. Ji C. M. Zheng Q. Huang Y. W. Wang B. (2023). Comparative analysis of newly identified rodent arteriviruses and porcine reproductive and respiratory syndrome virus to characterize their evolutionary relationships. Front. Vet. Sci. 10:1174031. doi: 10.3389/fvets.2023.1174031 37077949 PMC10106604

[ref63] Zhou H. Ren R. Yau S. S. (2023). Utilizing the codon adaptation index to evaluate the susceptibility to HIV-1 and SARS-CoV-2 related coronaviruses in possible target cells in humans. Front. Cell. Infect. Microbiol. 12:1085397. doi: 10.3389/fcimb.2022.1085397 36760235 PMC9905242

