# Supplementary material for: Comparative analysis of codon usage patterns of Plasmodium helical interspersed subtelomeric (PHIST) proteins
Source: Front Microbiol. 2023 Dec 14;14:1320060. doi: 10.3389/fmicb.2023.1320060 (PMC10752978; doi:10.3389/fmicb.2023.1320060)
Supplement: Supplementary file 1 [file Data_Sheet_1.DOCX]

**Table S1 Codon base composition in PHIST proteins**

| **Subgroup** | **ID** | **A3%** | **T3%** | **C3%** | **G3%** | **GC1%** | **GC2%** | **GC3%** | **GCs%** | **ATs%** |
| --- | --- | --- | --- | --- | --- | --- | --- | --- | --- | --- |
| **PHISTa** | PF3D7_0102000 | 62.99 | 61.86 | 13.08 | 4.64 | 32.81 | 22.13 | 13.44 | 22.79 | 77.21 |
|  | PF3D7_0115100 | 61.69 | 58.37 | 19.30 | 4.31 | 28.57 | 21.43 | 16.33 | 22.11 | 77.89 |
|  | PF3D7_0402000 | 65.52 | 50.58 | 17.73 | 10.04 | 39.39 | 23.78 | 20.51 | 27.89 | 72.11 |
|  | PF3D7_0424900 | 60.11 | 58.10 | 15.57 | 8.57 | 32.75 | 22.18 | 16.90 | 23.94 | 76.06 |
|  | PF3D7_0425300 | 63.58 | 56.25 | 12.88 | 6.25 | 30.90 | 24.03 | 15.88 | 23.61 | 76.39 |
|  | PF3D7_0425400 | 57.53 | 62.13 | 18.60 | 7.69 | 32.76 | 22.84 | 18.10 | 24.57 | 75.43 |
|  | PF3D7_0601700 | 64.89 | 56.89 | 9.04 | 6.67 | 34.14 | 27.59 | 13.10 | 24.94 | 75.06 |
|  | PF3D7_0800600 | 61.39 | 57.69 | 18.60 | 4.81 | 29.11 | 21.58 | 16.10 | 22.26 | 77.74 |
|  | PF3D7_0831750 | 70.50 | 56.94 | 7.95 | 4.31 | 33.22 | 20.42 | 9.34 | 20.99 | 79.01 |
|  | PF3D7_1000700 | 67.74 | 56.74 | 10.00 | 5.58 | 32.53 | 24.57 | 13.49 | 23.53 | 76.47 |
|  | PF3D7_1001100 | 63.21 | 57.46 | 12.57 | 7.89 | 35.03 | 22.93 | 15.29 | 24.42 | 75.58 |
|  | PF3D7_1001300 | 66.27 | 61.32 | 12.86 | 5.19 | 27.60 | 16.85 | 13.62 | 19.35 | 80.65 |
|  | PF3D7_1100600 | 68.36 | 58.96 | 7.79 | 6.13 | 32.27 | 23.40 | 11.70 | 22.46 | 77.54 |
|  | PF3D7_1149700 | 68.63 | 56.06 | 11.63 | 4.55 | 26.74 | 26.74 | 15.12 | 23.10 | 76.90 |
|  | PF3D7_1253100 | 66.31 | 65.35 | 12.42 | 1.98 | 26.99 | 20.42 | 11.07 | 19.49 | 80.51 |
|  | PF3D7_1253300 | 60.42 | 51.57 | 13.64 | 8.18 | 33.01 | 31.55 | 17.96 | 27.51 | 72.49 |
|  | PF3D7_1253800 | 65.61 | 57.35 | 11.73 | 9.31 | 33.33 | 20.00 | 15.09 | 22.81 | 77.19 |
|  | PF3D7_1253900 | 65.17 | 55.25 | 9.74 | 7.76 | 32.61 | 27.90 | 13.41 | 24.64 | 75.36 |
|  | PF3D7_1301100 | 64.44 | 56.42 | 10.83 | 6.88 | 33.81 | 27.70 | 13.31 | 24.94 | 75.06 |
|  | PF3D7_1301500 | 66.32 | 56.70 | 14.29 | 7.59 | 25.89 | 19.42 | 17.80 | 21.04 | 78.96 |
|  | PF3D7_1372000 | 69.57 | 55.00 | 10.80 | 8.00 | 35.17 | 24.88 | 14.35 | 24.80 | 75.20 |
|  | PF3D7_1400900 | 65.00 | 56.42 | 10.19 | 6.88 | 33.09 | 27.70 | 12.95 | 24.58 | 75.42 |
|  | PF3D7_1477700 | 50.00 | 57.76 | 17.72 | 11.64 | 35.27 | 24.32 | 22.26 | 27.28 | 72.72 |
|  | PF3D7_1478000 | 62.29 | 58.57 | 13.84 | 9.52 | 30.74 | 24.03 | 17.67 | 24.15 | 75.85 |
|  | PF3D7_1478500 | 64.12 | 57.00 | 11.49 | 3.38 | 32.45 | 27.17 | 13.21 | 24.28 | 75.72 |
|  | PF3D7_1479200 | 65.43 | 56.86 | 11.66 | 10.29 | 33.68 | 19.65 | 15.79 | 23.04 | 76.96 |
|  | PF3D7_1479300 | 64.44 | 56.42 | 10.83 | 6.88 | 33.81 | 27.70 | 13.31 | 24.94 | 75.06 |
| **PHISTa-like/PHIST** | PF3D7_0425250 | 72.41 | 53.72 | 14.29 | 7.44 | 29.12 | 15.38 | 15.93 | 20.15 | 79.85 |
|  | PF3D7_0831300 | 61.97 | 67.81 | 15.77 | 6.12 | 40.51 | 18.73 | 16.67 | 25.30 | 74.70 |
|  | PF3D7_0831500 | 66.35 | 61.42 | 10.75 | 4.33 | 30.60 | 23.51 | 11.61 | 21.73 | 78.27 |
|  | PF3D7_0831900 | 62.43 | 54.85 | 16.46 | 11.65 | 30.21 | 20.14 | 20.49 | 23.61 | 76.39 |
|  | PF3D7_0832200 | 65.96 | 58.45 | 9.94 | 3.65 | 36.93 | 25.78 | 11.85 | 24.85 | 75.15 |
|  | PF3D7_0832300 | 65.27 | 54.12 | 15.38 | 7.73 | 28.46 | 23.08 | 17.31 | 22.95 | 77.05 |
|  | PF3D7_0832700 | 63.82 | 55.77 | 13.28 | 7.69 | 32.95 | 22.22 | 16.86 | 24.01 | 75.99 |
|  | PF3D7_1201200 | 63.52 | 51.50 | 6.47 | 11.38 | 34.68 | 27.93 | 15.32 | 25.98 | 74.02 |
|  | PF3D7_1301300 | 65.36 | 60.00 | 11.03 | 8.33 | 30.65 | 23.79 | 14.52 | 22.98 | 77.02 |
|  | PF3D7_1372300 | 60.14 | 51.55 | 13.85 | 8.70 | 32.37 | 30.92 | 18.36 | 27.21 | 72.79 |
|  | PF3D7_1477300 | 46.49 | 56.05 | 21.08 | 11.66 | 33.45 | 30.60 | 24.56 | 29.54 | 70.46 |
|  | PF3D7_1477400 | 56.25 | 55.88 | 13.29 | 11.34 | 27.63 | 26.64 | 20.39 | 24.89 | 75.11 |
| **PHISTb** | PF3D7_0201600 | 64.17 | 56.20 | 15.71 | 9.22 | 34.57 | 22.84 | 19.14 | 25.51 | 74.49 |
|  | PF3D7_0401800 | 77.08 | 33.48 | 5.63 | 5.79 | 35.29 | 54.01 | 10.87 | 33.39 | 66.61 |
|  | PF3D7_0402100 | 64.16 | 58.26 | 12.21 | 9.86 | 38.37 | 20.14 | 17.19 | 25.23 | 74.77 |
|  | PF3D7_0424600 | 56.37 | 53.75 | 6.04 | 16.67 | 31.61 | 31.94 | 20.97 | 28.17 | 71.83 |
|  | PF3D7_0424800 | 62.02 | 50.81 | 15.22 | 9.68 | 31.11 | 24.44 | 18.73 | 24.76 | 75.24 |
|  | PF3D7_0532300 | 65.00 | 54.64 | 9.73 | 13.66 | 40.00 | 23.53 | 19.80 | 27.78 | 72.22 |
|  | PF3D7_0532400 | 64.67 | 56.77 | 10.21 | 5.99 | 37.05 | 28.17 | 13.61 | 26.28 | 73.72 |
|  | PF3D7_0601500 | 65.77 | 56.59 | 16.13 | 6.89 | 32.48 | 21.79 | 17.52 | 23.93 | 76.07 |
|  | PF3D7_0631100 | 65.55 | 56.76 | 16.13 | 6.61 | 32.33 | 21.84 | 17.34 | 23.84 | 76.16 |
|  | PF3D7_0702100 | 71.37 | 47.48 | 7.21 | 10.50 | 38.54 | 25.45 | 14.29 | 26.09 | 73.91 |
|  | PF3D7_0731300 | 61.21 | 60.34 | 11.98 | 4.74 | 31.45 | 24.53 | 14.47 | 23.48 | 76.52 |
|  | PF3D7_0831000 | 66.67 | 60.63 | 12.23 | 5.08 | 26.00 | 23.65 | 14.29 | 21.31 | 78.69 |
|  | PF3D7_0902700 | 63.12 | 60.73 | 10.71 | 9.57 | 40.53 | 25.39 | 16.70 | 27.54 | 72.46 |
|  | PF3D7_0936900 | 59.84 | 56.67 | 11.40 | 8.52 | 33.79 | 31.32 | 16.76 | 27.29 | 72.71 |
|  | PF3D7_0937000 | 61.43 | 60.74 | 13.26 | 8.89 | 27.93 | 20.67 | 17.88 | 22.16 | 77.84 |
|  | PF3D7_1102500 | 59.58 | 58.71 | 13.65 | 7.14 | 35.52 | 24.64 | 16.96 | 25.71 | 74.29 |
|  | PF3D7_1201000 | 65.45 | 61.36 | 12.32 | 10.30 | 46.33 | 17.89 | 15.75 | 26.66 | 73.34 |
|  | PF3D7_1252700 | 59.54 | 56.77 | 12.96 | 6.63 | 33.48 | 29.93 | 16.19 | 26.53 | 73.47 |
|  | PF3D7_1252800 | 63.71 | 63.93 | 13.07 | 5.83 | 28.71 | 20.10 | 14.83 | 21.21 | 78.79 |
|  | PF3D7_1372100 | 62.50 | 55.66 | 18.69 | 8.82 | 33.44 | 18.52 | 20.16 | 24.04 | 75.96 |
|  | PF3D7_1401600 | 60.12 | 56.69 | 12.50 | 7.85 | 38.41 | 29.65 | 18.16 | 28.74 | 71.26 |
|  | PF3D7_1476200 | 63.77 | 58.58 | 16.61 | 11.83 | 36.65 | 21.64 | 19.69 | 25.99 | 74.01 |
|  | PF3D7_1476300 | 67.01 | 59.11 | 13.29 | 6.25 | 37.52 | 17.98 | 15.36 | 23.62 | 76.38 |
|  | PF3D7_1477500 | 61.45 | 55.11 | 16.14 | 9.09 | 36.93 | 23.55 | 18.76 | 26.41 | 73.59 |
| **PHISTb-DnaJ** | PF3D7_0102200 | 65.38 | 53.48 | 7.63 | 12.57 | 49.72 | 24.40 | 16.30 | 30.14 | 69.86 |
|  | PF3D7_0201700 | 65.35 | 57.52 | 12.62 | 6.43 | 38.07 | 21.31 | 15.21 | 24.86 | 75.14 |
|  | PF3D7_0220100 | 62.27 | 56.28 | 12.39 | 10.47 | 33.67 | 22.95 | 18.34 | 24.98 | 75.02 |
|  | PF3D7_1038800 | 66.10 | 56.72 | 12.03 | 6.20 | 31.36 | 24.67 | 15.13 | 23.72 | 76.28 |
|  | PF3D7_1149200 | 66.54 | 53.24 | 10.16 | 9.19 | 45.28 | 23.83 | 15.67 | 28.26 | 71.74 |
|  | PF3D7_1149500 | 64.78 | 50.90 | 10.89 | 12.11 | 33.69 | 24.88 | 17.86 | 25.48 | 74.52 |
|  | PF3D7_1201100 | 65.00 | 56.17 | 11.49 | 6.33 | 33.52 | 26.42 | 16.32 | 25.42 | 74.58 |
| **PHISTc** | PF3D7_0202100 | 63.73 | 58.56 | 10.92 | 4.50 | 30.36 | 29.70 | 16.17 | 25.41 | 74.59 |
|  | PF3D7_0219700 | 60.47 | 57.59 | 10.34 | 14.29 | 28.38 | 21.62 | 20.95 | 23.65 | 76.35 |
|  | PF3D7_0219800 | 55.72 | 53.02 | 14.63 | 18.97 | 27.50 | 23.12 | 26.25 | 25.62 | 74.38 |
|  | PF3D7_0424000 | 56.77 | 54.14 | 15.87 | 12.78 | 34.48 | 28.16 | 21.26 | 27.97 | 72.03 |
|  | PF3D7_0532200 | 60.00 | 55.56 | 14.52 | 12.35 | 30.84 | 26.87 | 22.91 | 26.87 | 73.13 |
|  | PF3D7_0731100 | 60.65 | 58.14 | 11.70 | 7.12 | 37.82 | 33.67 | 15.38 | 28.96 | 71.04 |
|  | PF3D7_0801000 | 64.23 | 56.66 | 12.80 | 7.99 | 41.15 | 29.59 | 16.72 | 29.15 | 70.85 |
|  | PF3D7_0830600 | 65.48 | 58.79 | 12.45 | 6.63 | 35.23 | 22.54 | 14.88 | 24.22 | 75.78 |
|  | PF3D7_0936600 | 54.00 | 54.51 | 18.64 | 8.58 | 28.86 | 29.19 | 20.81 | 26.29 | 73.71 |
|  | PF3D7_0936800 | 72.38 | 50.62 | 8.15 | 8.30 | 43.49 | 25.78 | 14.06 | 27.78 | 72.22 |
|  | PF3D7_1001700 | 65.73 | 62.98 | 10.13 | 5.52 | 38.58 | 22.10 | 13.11 | 24.59 | 75.41 |
|  | PF3D7_1001800 | 64.80 | 60.87 | 8.28 | 5.43 | 32.95 | 26.44 | 12.26 | 23.88 | 76.12 |
|  | PF3D7_1016500 | 60.79 | 62.11 | 15.17 | 8.77 | 28.26 | 23.05 | 17.84 | 23.05 | 76.95 |
|  | PF3D7_1016600 | 62.89 | 66.36 | 9.45 | 10.00 | 28.62 | 17.85 | 17.17 | 21.21 | 78.79 |
|  | PF3D7_1016700 | 59.38 | 65.98 | 12.07 | 6.96 | 32.25 | 26.23 | 15.52 | 24.67 | 75.33 |
|  | PF3D7_1016800 | 71.30 | 57.59 | 13.50 | 3.66 | 35.24 | 21.27 | 15.87 | 24.13 | 75.87 |
|  | PF3D7_1148700 | 75.20 | 45.28 | 7.83 | 16.54 | 33.42 | 26.42 | 18.87 | 26.24 | 73.76 |
|  | PF3D7_1200900 | 63.21 | 67.60 | 13.89 | 4.53 | 30.73 | 20.05 | 14.32 | 21.70 | 78.30 |

Note：Single underline represents the minimum value; Double underline represents maximum value.
